# Soy Protein and Safflower-Seed Oil Attenuate Inflammation and Immune Dysfunction in Rats with Hyperuricemia

**DOI:** 10.3390/ijms252312977

**Published:** 2024-12-03

**Authors:** Yi-Fang Liu, Yi-Chen Wu, Yu Yang, Hui-Chen Lo

**Affiliations:** 1 Department of Nutritional Science, Fu Jen Catholic University, New Taipei City 242062, Taiwan; 070647@mail.fju.edu.tw; 2 Department of Food and Nutrition, Tri-Service General Hospital, Taipei 114202, Taiwan; yichen8019@mail.ndmctsgh.edu.tw; 3 Division of Nephrology, Internal Medicine, Changhua Christian Hospital, Changhua 500209, Taiwan; 2219@cch.org.tw

**Keywords:** hyperuricemia, casein, soy protein, palm oil, safflower-seed oil, immunocyte subsets, cytokine

## Abstract

A plant-based diet is considered a promising approach for managing hyperuricemia (HUA). This study examined the effects of soy protein and plant-based oils on HUA-induced inflammation and immune dysfunction. Male Wistar rats, induced with HUA using oxonic acid and uric acid (UA), were fed casein or soy protein with palm or safflower oil (2 × 2 factorial design) for 8 weeks. HUA rats had lower serum albumin and T cell percentages in peripheral blood leukocytes (PBLs) and splenocytes, along with increased leukocyte counts and spleen weights, compared to healthy rats (*p* < 0.05). Soy protein improved HUA-induced reductions in albumin, while safflower-seed oil ameliorated reductions in albumin, plasma interleukin (IL)-4, and T-suppressor splenocytes, and mitigated elevated serum UA, plasma IL-6, and B leukocytes (two-way ANOVA, *p* < 0.05). In PBL, soy protein alleviated HUA-induced decreases in TNF-α, casein and palm oil increased IL-6, and casein further reduced IFN-γ production. Under Con A stimulation, casein and safflower-seed oil alleviated decreases in IL-6 and IL-10, respectively, while under LPS stimulation, casein further increased TNF-α production. In splenocytes, soy protein and safflower-seed oil reduced HUA-induced increases in TNF-α and increased IL-10, and safflower-seed oil increased IL-6 production. Under Con A stimulation, soy protein and safflower-seed oil reduced TNF-α and increased IL-10 production in splenocytes. The findings suggest that soy protein and safflower-seed oil may counteract HUA-related inflammation, alleviate monocyte activation, and enhance Th2 immune response in HUA. A plant-based diet rich in soy protein and safflower-seed oil may help manage HUA and associated inflammation and immune dysfunction.

## 1. Introduction

Hyperuricemia (HUA), characterized by serum uric acid (UA) levels exceeding 70 mg/L in men and 60 mg/L in women, is a growing global health issue due to its association with obesity, hypertension, dyslipidemia, type 2 diabetes, chronic kidney disease, cardiovascular disease, and metabolic syndrome [1]. These conditions are driven by oxidative stress, inflammation, and endothelial dysfunction linked to HUA [1]. The prevalence of HUA is notably high in affluent countries, affecting up to 20.1% of the U.S. population [2] and 16.4% in China [3], posing a significant public health burden. Epidemiological studies show that factors such as male gender, aging, genetics, obese, smoking, and sedentary behavior are positively associated with HUA [4].

Diet also plays a crucial role in HUA risk, with animal-based and sweet food patterns positively correlated and plant-based food diets (e.g., vegetarian) inversely correlated with HUA [5,6]. Soy and its products, moderately high in purines, are a major plant protein source in Asian countries, with growing consumption in Western nations [7]. Palm oil, rich in saturated fatty acids and used as a replacement for trans-fatty acid-rich hydrogenated oils, is a leading cooking oil globally [8]. In our previous study using oxonic acid (OA)- and UA-induced HUA rats, we found that casein with safflower-seed oil, rich in polyunsaturated fatty acids (PUFAs), may attenuate HUA-associated renal damage, while soy protein with safflower-seed oil may lower serum UA and triglycerides [9].

Evidence suggests that elevated UA levels may trigger a strong inflammatory response by increasing the production of reactive oxygen species (ROS), activating innate immunity, and affecting T cell proliferation and function, thereby accelerating the progression of HUA, disrupting immune function, and causing further damage to distant organs, such as the kidneys and the heart [10]. Dietary modifications, especially plant-based diets, may be a potential way to improve HUA-associated inflammation and immune dysregulation [11]. For example, casein, soy protein, and their derivatives have been reported to exhibit antioxidant and anti-inflammatory properties by modulating immune responses [12,13]. Furthermore, intravenous injection of n-3 PUFAs has been shown to improve immune function and reduce inflammatory responses by decreasing CD3+ and CD4+ cells while increasing IL-4 secretion and CD8+ cells in patients with hypertension [14].

Understanding immune cell changes in HUA and its complications is critical for gaining insights into disease progression and developing effective treatment strategies. In this study, we aimed to evaluate the effects of milk- and plant-based proteins (casein and soy protein) and oils (palm oil and safflower-seed oil) on immune function in HUA. We hypothesized that plant-based diets may attenuate HUA and its complications by improving HUA-related immune dysfunction. To test this, we analyzed immune cell profiles and cytokine production, with or without mitogen stimulation, in the peripheral blood leukocytes (PBLs) and splenocytes.

## 2. Results

### 2.1. Body Weight and Body Weight Gain

**Figure 1:**
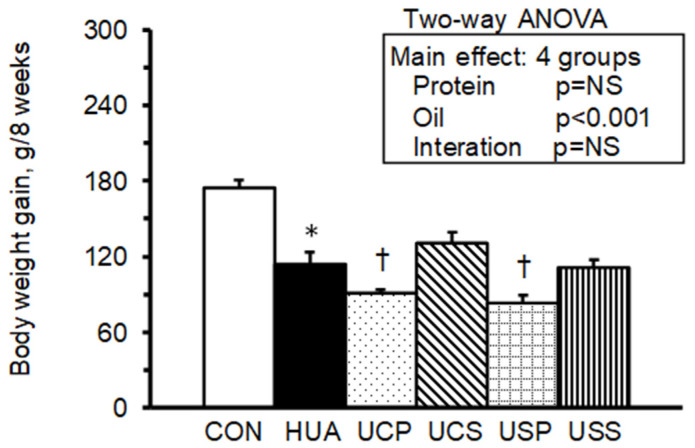
Body weight gain is calculated as the difference between body weight at week 8 and week 0. Values are means ± SEM, *n* = 8 per group. *, HUA vs. CON; †, UCP, UCS, USP, and USS vs. HUA (one-way ANOVA with least significant difference, *p* < 0.05). Values of a two-way ANOVA are *p*-values for main effects and interactions of protein (casein or soy protein) and oil (palm or safflower-seed oil) in the UCP, UCS, USP, and USS groups. NS, not significant.

Initial body weights at week 0 ranged from 209 to 212 g, with no significant differences among the groups. By week 8, the hyperuricemic (HUA) group, fed casein and corn oil as part of the AIN-93M diet, had a significantly lower body weight (319 ± 10 g) compared to the normal control (CON) group (383 ± 9 g). The USP group, hyperuricemic rats fed soy protein isolate and palm oil, exhibited an even lower body weight (291 ± 5 g) compared to the HUA group. Body weight gain from week 0 to week 8 was significantly lower in the HUA group compared to the CON group and further reduced in the UCP (hyperuricemic rats fed casein and palm oil) and USP groups compared to the HUA group (Figure 1). A two-way analysis of variance (ANOVA) indicated that the oil source was the main factor influencing body weight gain, with palm oil significantly reducing body weight gain.

### 2.2. Serum Biochemical Values, Immunocyte Counts, and Spleen Weights

Serum albumin levels were significantly lower in the HUA group compared to the CON group but were significantly increased in the USS group, consisting of hyperuricemic rats fed soy protein isolate and safflower-seed oil, compared to the HUA group. Uric acid levels were significantly higher in the HUA group compared to the CON group but were significantly reduced in the UCS (hyperuricemic rats fed casein and safflower-seed oil) and USS groups (Table 1). Serum glucose and triglyceride levels did not show significant differences between the CON and HUA groups. However, triglyceride levels were significantly elevated in the UCP and USP groups and reduced in the UCS and USS groups compared to the HUA group. A two-way ANOVA revealed that protein and oil sources were key factors influencing serum albumin levels, with soy protein and safflower-seed oil increasing albumin in the UCP, UCS, USP, and USS groups. Additionally, the oil source played a significant role in influencing serum glucose, triglyceride, and uric acid levels, with safflower-seed oil reducing triglyceride and uric acid and palm oil increasing glucose and triglyceride levels.

Leukocyte counts and spleen weights were significantly higher in the HUA group compared to the CON group, with no significant differences observed among the HUA, UCP, UCS, USP, and USS groups. Splenocyte counts did not differ significantly across all groups. A two-way ANOVA indicated that the oil source was the main factor affecting leukocyte and splenocyte counts and spleen weight, with safflower-seed oil reducing leukocyte counts and spleen weight, and palm oil reducing splenocyte counts in rats with HUA.

### 2.3. Plasma Cytokine Concentrations

**Figure 2:**
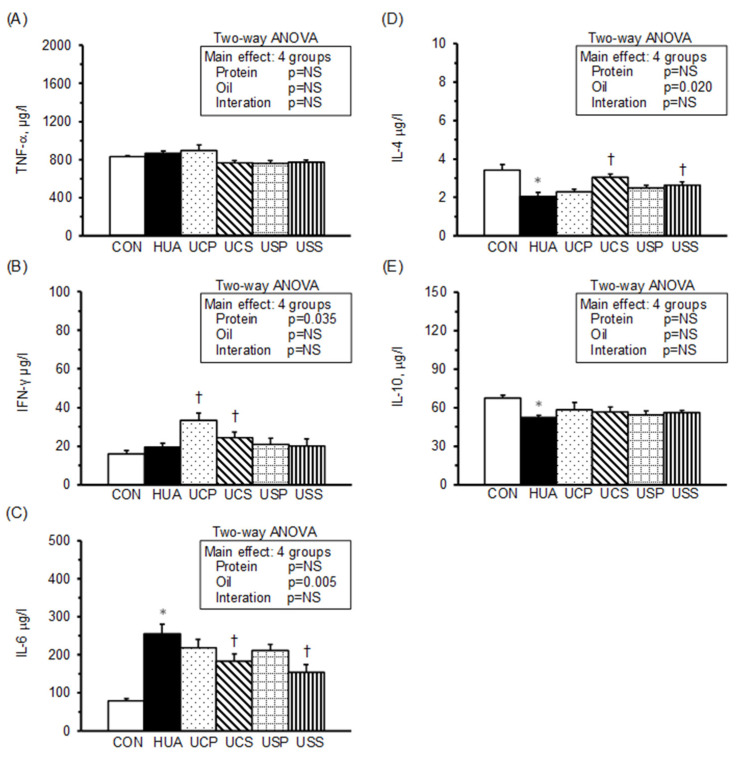
Plasma cytokine concentrations. TNF-α (**A**), IFN-γ (**B**), IL-4 (**C**), IL-6 (**D**), and IL-10 (**E**). Values are means ± SEM, *n* = 8 per group. *, HUA vs. CON; †, UCP, UCS, USP, and USS vs. HUA (one-way ANOVA with least significant difference, *p* < 0.05). Values of a two-way ANOVA are *p*-values for main effects and interactions of protein (casein or soy protein) and oil (palm or safflower-seed oil) in the UCP, UCS, USP, and USS groups. NS, not significant.

Plasma levels of tumor necrosis factor (TNF)-α, interferon (IFN)-γ, interleukin (IL)-6, IL-4, and IL-10 are shown in Figure 2. TNF-α levels did not differ significantly across all groups (Figure 2A), nor did IFN-γ levels between the HUA and CON groups (Figure 2B). However, the HUA group exhibited significantly higher IL-6 levels (Figure 2C) and lower IL-4 and IL-10 levels (Figure 2D,E) compared to the CON group. Among the hyperuricemic groups, the UCP and UCS groups had significantly elevated IFN-γ levels, while the UCS and USS groups had significantly lower IL-6 and higher IL-4 levels compared to the HUA group.

A two-way ANOVA indicated that neither protein nor oil source were the main factors affecting plasma TNF-α and IL-10 levels. However, the protein source significantly influenced plasma IFN-γ levels, with palm oil increasing IFN-γ in hyperuricemic rats. The oil source was the main factor affecting plasma IL-6 and IL-4 levels, with safflower-seed oil reducing IL-6 and elevating IL-4 levels in hyperuricemic rats. Additionally, there was no significant interaction between protein and oil sources affecting these cytokines in plasma.

### 2.4. Subpopulation Distribution of Immunocytes in Peripheral Blood and Spleen

The distribution of leukocyte subsets in peripheral blood is presented in Table 2. The HUA group showed significantly reduced percentages of total-T cells (CD3+), helper T cells (CD3+CD4+), and suppressor T cells (CD3+CD8b+) and significantly elevated percentages of B cells (CD45RA+IgM+) compared to the CON group. No significant differences were observed in the percentages of monocytes (CD11b/c+GN−), granulocytes (CD11b/c+GN+), and natural killer cells (NKR+CD3−) between the HUA and CON groups. Among the hyperuricemic groups, the UCS, USP, and USS groups exhibited significantly decreased percentages of B cells, with the UCS group showing significantly increased monocyte percentages, and the USP and USS groups showing significantly decreased percentages of natural killer cells compared to the HUA group.

A two-way ANOVA revealed that neither protein nor oil sources were the main factors affecting the distribution of total T, helper T, suppressor T, or natural killer cells in the peripheral blood of hyperuricemic rats. However, the oil source significantly influenced B cell and monocyte percentages, with safflower-seed oil decreasing B cell percentages and increasing monocyte percentages. A significant interaction was found between protein and oil sources affecting monocyte percentages, where soy protein mitigated the monocyte-elevating effect of safflower-seed oil. The protein source was the main factor affecting granulocyte percentages, with soy protein reducing granulocyte percentages in the peripheral blood of hyperuricemic rats.

The distribution of splenocyte subsets is shown in Table 3. The HUA group exhibited significantly lower percentages of total T cells and suppressor T cells compared to the CON group, with no significant differences observed in helper T cells, B cells, macrophages, granulocytes, or natural killer cells. Among the hyperuricemic rats, the USS group showed significantly increased percentages of total T cells, suppressor T cells, and granulocytes, and decreased macrophage percentages compared to the HUA group. Additionally, the UCP and USP groups showed significantly reduced percentages of natural killer cells compared to the HUA group.

A two-way ANOVA revealed that neither protein nor oil sources were the main factors affecting the distribution of helper T cells or granulocytes in the splenocytes of hyperuricemic rats. However, significant interactions between protein and oil sources were observed in the percentages of total T cells and macrophages, with soy protein and safflower-seed oil synergistically elevating total T cell percentages and reducing macrophage percentages. The oil source significantly influenced suppressor T cell and natural killer cell percentages, with safflower-seed oil increasing suppressor T cell percentages and palm oil decreasing natural killer cell percentages. The protein source was the main factor affecting B cell percentages, with casein reducing B cell percentages in the splenocytes of hyperuricemic rats.

### 2.5. Splenocyte Proliferation

**Figure 3:**
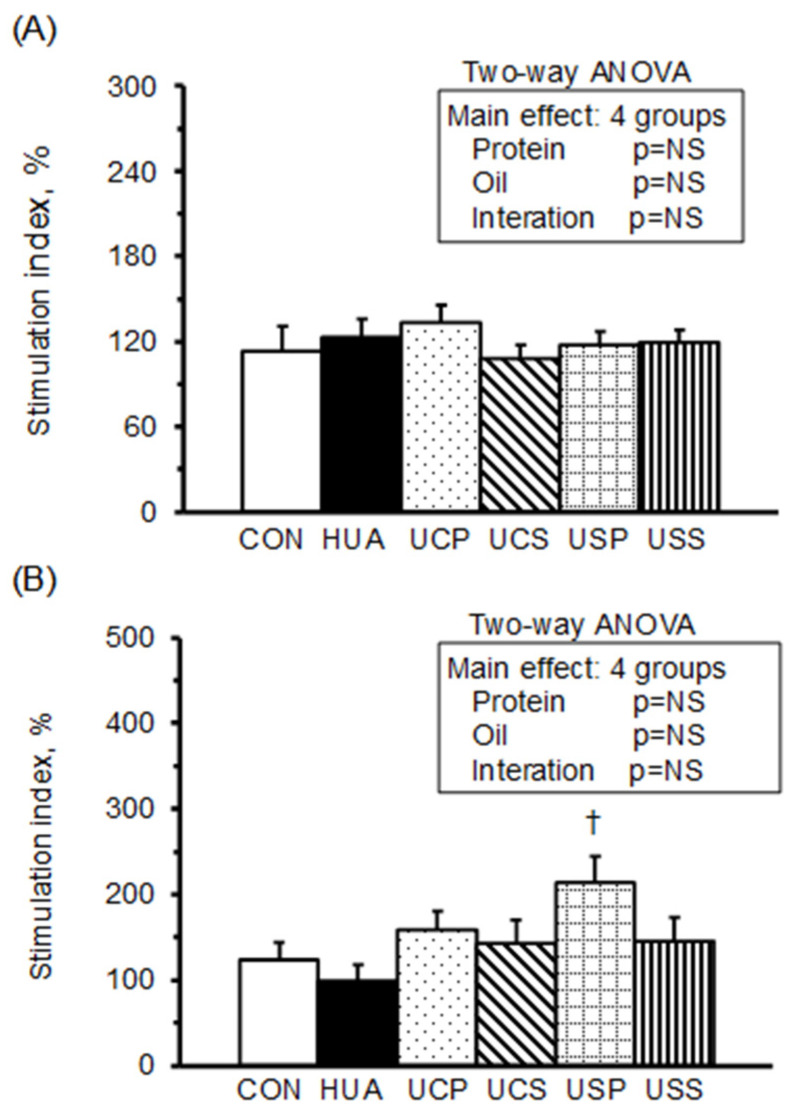
Cell proliferation of splenocytes. Splenocytes were cultured with RPMI 1640 medium and simulated with Con A (**A**) or LPS (**B**). The stimulation index was calculated from OD values (at 490 nm) of splenocytes cultured with Con A or LPS divided by those with RPMI 1640 medium and multiplied by 100. Values are means ± SEM, *n* = 8 per group. †, UCP, UCS, USP, and USS vs. HUA (one-way ANOVA with least significant difference, *p* < 0.05). Values of a two-way ANOVA are *p*-values for main effects and interactions of protein (casein or soy protein) and oil (palm or safflower-seed oil) in the UCP, UCS, USP, and USS groups. NS, not significant.

The stimulating indices (SI) of splenocyte proliferation, calculated from the optical density of splenocyte cultures in RPMI-1640 medium with mitogen concanvavalin A (Con A) or lipopolysaccharide (LPS) relative to RPMI-1640 medium alone, are presented in Figure 3. The stimulation index of splenocyte proliferation with Con A or LPS did not differ significantly between the HUA and CON groups. However, among the hyperuricemic rats, the USP group exhibited a significantly higher stimulation index with LPS stimulation. A two-way ANOVA revealed that neither protein nor oil source had a significant effect on splenocyte proliferation with Con A or LPS stimulation.

### 2.6. Cytokine Production of Peripheral Blood Leukocytes (PBLs)

**Figure 4:**
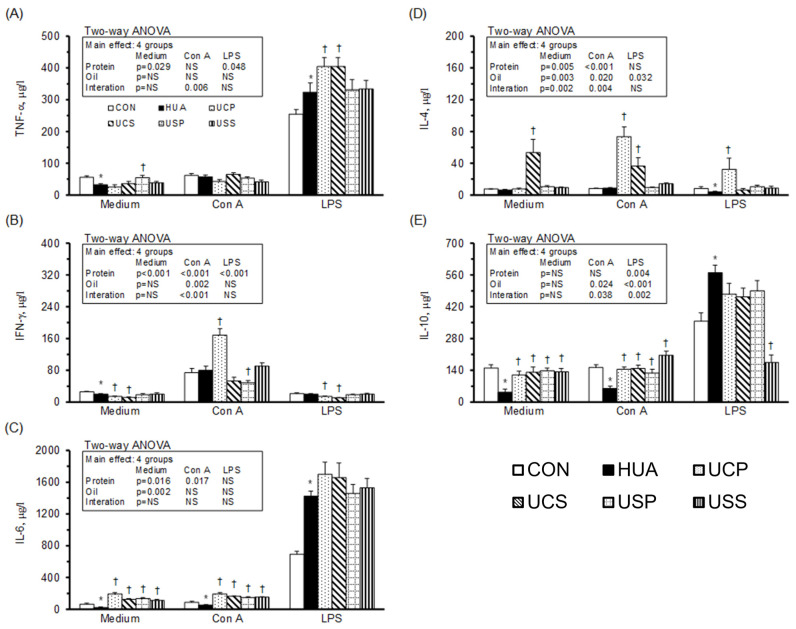
Cytokine production of leukocytes. TNF-α (**A**), IFN-γ (**B**), IL-6 (**C**), IL-4 (**D**), and IL-10 (**E**) productions from leukocytes cultured in RPMI-1640 medium, either unstimulated or stimulated with Con A and LPS. Values are means ± SEM, *n* = 8 per group. *, HUA vs. CON; †, UCP, UCS, USP, and USS vs. HUA (one-way ANOVA with least significant difference, *p* < 0.05). Values of a two-way ANOVA are *p*-values for main effects and interactions of protein (casein or soy protein) and oil (palm or safflower-seed oil) in the UCP, UCS, USP, and USS groups. NS, not significant.

Figure 4 illustrates the cytokine production of TNF-α, IFN-γ, IL-6, IL-4, and IL-10 in PBLs cultured in RPMI-1640 medium, either unstimulated or stimulated with Con A and LPS. In the unstimulated medium, the production of TNF-α (Figure 4A), IFN-γ (Figure 4B), IL-6 (Figure 4C), and IL-10 (Figure 4E) was significantly lower in the HUA group compared to the CON group. The USP group showed an increase in TNF-α, while the UCP and UCS groups exhibited further reductions in IFN-γ. Additionally, the UCP, UCS, USP, and USS groups had increased IL-6 and IL-10 production compared to the HUA group. A two-way ANOVA identified the protein source as the primary factor influencing TNF-α, IFN-γ, IL-6, and IL-4 (Figure 4D) production in the unstimulated medium, with soy protein enhancing TNF-α and casein reducing IFN-γ while increasing IL-4 and IL-6 production in hypouricemic rats. Furthermore, safflower-seed oil and palm oil were the main factors driving increased IL-4 and IL-6 production, respectively.

Under Con A stimulation, the production of IL-6 and IL-10 was significantly reduced in the HUA group compared to the CON group but increased in the UCP, UCS, USP, and USS groups compared to the HUA group. The UCP group showed increased IFN-γ and IL-4 levels, the UCS group had increased IL-4, and the USP group exhibited reduced IFN-γ compared to the HUA group. A two-way ANOVA indicated that casein increased IL-6 and IL-4, soy protein decreased IFN-γ and increased IL-6, palm oil increased IFN-γ, IL-4, and IL-10, and safflower-seed oil increased IL-10 in the PBLs of hypouricemic rats.

Under LPS stimulation, the production of TNF-α, IL-6, and IL-10 was significantly increased, while IL-4 production was significantly decreased in the HUA group compared to the CON group. The UCP and UCS groups showed further increases in TNF-α and decreases in IFN-γ, the UCP group had increased IL-4, and the USS group had decreased IL-10 production compared to the HUA group. A two-way ANOVA revealed that casein was the primary factor increasing TNF-α and decreasing IFN-γ, palm oil was the main factor increasing IL-4, and soy protein and safflower-seed oil were the key factors decreasing IL-10 in the PBLs of hypouricemic rats.

### 2.7. Cytokine Production of Splenocytes

**Figure 5:**
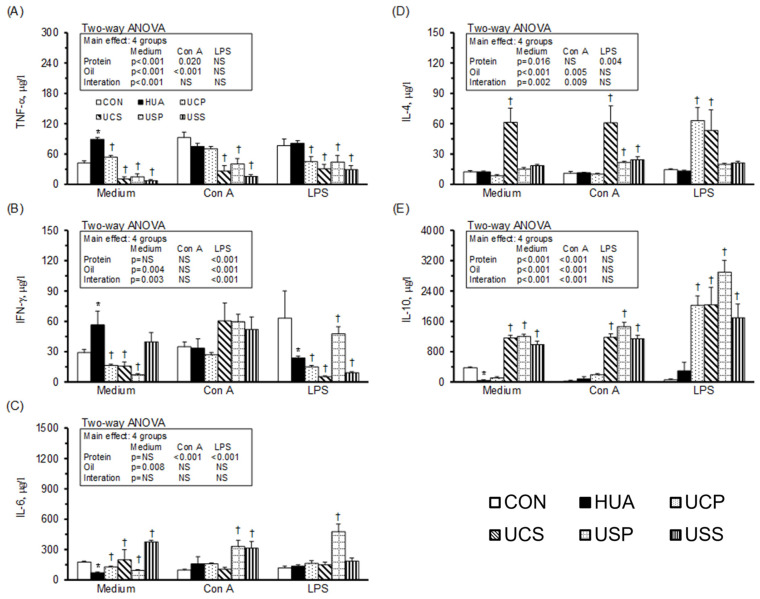
Cytokine production of splenocytes. TNF-α (**A**), IFN-γ (**B**), IL-6 (**C**), IL-4 (**D**), and IL-10 (**E**) productions from leukocytes cultured in RPMI-1640 medium, either unstimulated or stimulated with Con A and LPS. Values are means ± SEM, *n* = 8 per group. *, HUA vs. CON; †, UCP, UCS, USP, and USS vs. HUA (one-way ANOVA with least significant difference, *p* < 0.05). Values of a two-way ANOVA are *p*-values for main effects and interactions of protein (casein or soy protein) and oil (palm or safflower-seed oil) in the UCP, UCS, USP, and USS groups. NS, not significant.

Figure 5 illustrates cytokine production in splenocytes. In the unstimulated medium, the production of TNF-α (Figure 5A) and IFN-γ (Figure 5B) was significantly higher, while IL-6 (Figure 5C) and IL-10 (Figure 5E) were significantly lower in the HUA group compared to the CON group. The UCP, UCS, USP, and USS groups showed decreases in TNF-α and increases in IL-6, while the UCP, UCS, and USP groups exhibited reduced IFN-γ. The UCS, USP, and USS groups showed increased IL-10, and the UCS group had increased IL-4 (Figure 5D) compared to the HUA group. A two-way ANOVA revealed that both protein and oil sources were the main factors in decreasing TNF-α and increasing IL-10, with soy protein and safflower-seed oil having stronger impacts than casein and palm oil. Additionally, palm oil was the stronger factor in decreasing IFN-γ production compared to safflower-seed oil, casein was the factor increasing IL-4, and safflower-seed oil was the factor increasing IL-6 production in the splenocytes of hyperuricemic rats.

Under Con A stimulation, the UCS, USP, and USS groups showed significantly decreased TNF-α and increased IL-4 and IL-10, while the USP and USS groups had significantly increased IL-6 production compared to the HUA group. A two-way ANOVA revealed that soy protein and safflower-seed oil had stronger impacts than casein and palm oil in decreasing TNF-α and increasing IL-10. Additionally, soy protein and safflower-seed oil were the main factors in increasing IL-6 and IL-4 production, respectively, in the splenocytes of hyperuricemic rats.

Under LPS stimulation, IFN-γ was significantly lower in the HUA group compared to the CON group, while the UCP, UCS, USP, and USS groups showed significantly decreased TNF-α, further reduced IFN-γ, and significantly increased IL-10 production compared to the HUA group. Additionally, the UCP and UCS groups had increased IL-4 and the USP group had increased IL-6 production compared to the HUA group. A two-way ANOVA indicated that casein and safflower-seed oil were the main factors in decreasing IFN-γ, casein in increasing IL-4, and soy protein in increasing IL-6 production in the splenocytes of hyperuricemic rats.

## 3. Discussion

HUA-induced immune dysfunction and systemic inflammation are linked to metabolic syndrome and various forms of organ damage [10]. In our previous study, we demonstrated that oral administration of soy protein or casein combined with safflower-seed oil attenuated HUA and HUA-induced renal damage [9]. Expanding on these findings, this study explored whether a plant-based diet, particularly one including soy protein and safflower-seed oil, can counteract HUA-related immune dysfunction and inflammation, potentially offering a nutritional strategy for managing HUA and its complications.

Evidence shows that high levels of soluble UA have pro-inflammatory and pro-oxidative effects [10]. However, findings on HUA-induced immune changes remain controversial. A retrospective study found that individuals with asymptomatic HUA had increased peripheral CD4+ T cells, along with higher serum IL-4 and IL-10 levels, indicating a Th2-dominant immune status [15]. In contrast, soluble UA (not monosodium urate crystals) inhibited CD14+ monocytes’ inflammatory function under LPS stimulation, suggesting suppressed innate immunity [16]. This discrepancy of immune responses may be due to the formation of UA crystals. In this study, HUA rats showed lower body weight gain, reduced serum albumin, higher leukocyte counts, and increased spleen weights, suggesting a catabolic and inflammatory status. Elevated plasma IL-6, along with decreased plasma IL-4 and IL-10, indicated heightened pro-inflammatory and suppressed anti-inflammatory responses in the circulation.

To investigate the effects of HUA on immune function, we analyzed PBLs and splenocytes to assess systemic and lymphoid organ-derived immune statuses. PBLs reflect circulating immune cells and immediate responses, while splenocytes provide insights into adaptive immunity and antigen-specific responses. HUA rats exhibited lower T cell percentages, particularly T-suppressor cells, in both PBLs and splenocytes, and higher B cell percentages in PBLs, indicating impaired T cell-mediated regulation. Pro-inflammatory cytokines (TNF-α and IFN-γ) were reduced in leukocytes but elevated in splenocytes, with decreased anti-inflammatory IL-10 in both, suggesting compartmentalized immune dysfunction and a shift in immune activation to lymphoid organs. Increased splenocyte cytokine production reflects heightened immune activity, driven by chronic inflammation and oxidative stress, likely mediated by UA via inflammasome activation [16] Additionally, LPS-activated leukocytes produced more TNF-α and IL-6 but less IL-4, indicating a pro-inflammatory skew with suppressed Th2-related activity. These findings reveal that HUA rats exhibit immune dysfunction characterized by altered cell proportions and dysregulated cytokine production.

Animal studies indicate that milk- and soy-derived proteins have immunomodulatory effects [12,13]. For example, β-casein and casein hydrolysate improved gut microbiota, enhanced splenocyte proliferation, macrophage phagocytic capacity, and NK cell activity, and reduced serum TNF-α and IL-1β in immunosuppressive mice and low-protein-fed pigs [17,18]. Soy protein isolate exhibited antioxidant, anti-inflammatory, and immunomodulatory properties, increasing serum immunoglobulins and reduced TNF-α and IL-1β in burn-stress aged rats [19], while mitigating LPS-induced ROS and NO secretion in macrophages [20]. However, their effects in HUA remained unexplored. This study demonstrated that casein ingestion in HUA rats enhanced IFN-γ reduction in PBLs, alleviated IL-6 reduction and increased IL-4 secretion in T-leukocytes, while reducing TNF-α elevation and increasing IL-10 secretion in T-splenocytes. Soy protein alleviated TNF-α reduction in leukocytes and T-splenocytes, increased IL-6 in T-leukocytes and macrophages, and balanced cytokine levels by reducing TNF-α in T-splenocytes and increasing IL-10 in T-splenocytes. These findings suggest that both proteins improve anti-inflammatory status and might activate the Th2 response in HUA rats, with soy protein showing superior effects on macrophages and T-splenocytes.

Palm oil, rich in saturated fatty acids, and safflower oil, high in PUFAs, have distinct fatty acid compositions influencing health. The World Health Organization reports that palm oil consumption may raise total cholesterol, LDL cholesterol, and the risk of non-fatal acute myocardial infarction compared to unsaturated vegetable oils [21]. In contrast, PUFA-rich oils like safflower oil may reduce coronary events by lowering lipid levels [22]. N-3 PUFAs, such as eicosapentaenoic acid and docosahexaenoic acids from fish oil, are known for antioxidant, anti-inflammatory, and immunomodulatory effects, including reduced T-lymphocyte proliferation and cytokine production [23]. However, plant oils’ effects on immune function in HUA were previously unexamined. This study found palm oil increased pro-inflammatory cytokines in T-leukocytes, while safflower-seed oil reduced serum UA, IL-6, and TNF-α, and increased IL-10, highlighting its superior anti-inflammatory effects in mitigating HUA-induced inflammation.

A plant-based dietary pattern may improve health, particularly in hyperlipidemia and cardiovascular disease, while being environmentally sustainable. However, the quality of plant-based components is crucial for optimal benefits [24]. This study showed that combining soy protein with safflower-seed oil, rather than palm oil, mitigated reductions in serum albumin and plasma IL-4 (a Th2 cytokine) while alleviating elevations in serum UA and plasma IL-6 (an innate immune cytokine) [15]. These findings suggest anabolic, anti-HUA, and anti-inflammatory effects in HUA rats. Additionally, changes in immune cell profiles—reduced B cells and granulocytes in PBLs, along with decreased macrophages and increased total T cells and T-suppressor cells in splenocytes—indicate a shift toward innate to adaptive immunity.

Additionally, HUA-induced decreases in IL-6 and IL-10, and T cell-secreted cytokines [15] were alleviated by all combinations of casein or soy protein with palm or safflower-seed oil in PBLs cultured with medium alone or under Con A stimulation, indicating an activated Th2 immune response. However, casein combined with wither palm or safflower-seed oil increased TNF-α production under LPS stimulation, suggesting an activated monocyte response. In splenocytes, TNF-α production decreased under medium alone or LPS stimulation, indicating reduced macrophage activity, while IL-4 and IL-10 increased under Con A stimulation, reflecting Th2 activation [25]. This effect was most pronounced with soy protein and safflower-seed oil. These changes suggest that a diet combining soy protein and safflower-seed oil may suppress innate immunity and enhance Th2 responses in HUA.

Significant interactions between protein and oil sources influenced immune profile and functions in HUA rats, likely through mechanisms involving oxidative stress, lipid metabolism, and amino acid composition. Soy protein reduced the monocyte-elevating effect of safflower-seed oil, resulting in lower macrophage percentages (innate immunity) and higher total T splenocyte percentages (adaptive immunity), demonstrating their interplay in balancing immune responses. In PBLs stimulated with Con A, soy protein diminished the palm-oil-induced elevation in IFN-γ and IL-4 while enhancing safflower-seed-oil-induced IL-10 production, indicating anti-inflammatory synergy. In splenocytes, casein’s effects varied with the oil source: it reduced the IL-10-elevating effects of palm-oil, suggesting diminished anti-inflammatory activity, while amplifying safflower-seed oil-induced IL-4 production, promoting Th2-related responses. Soy protein also mitigated safflower-seed-oil-induced IFN-γ increases, modulating pro-inflammatory adaptive immunity in HUA rats. These findings highlight the critical role of dietary combinations in regulating innate and adaptive immunity in HUA.

This study has several limitations. First, we focused on casein versus soy protein and palm oil versus safflower-seed oil, limiting generalizability to other dietary proteins and oils. Thus, the findings provide only a partial view of how plant-based diets may affect immune responses in HUA. Second, we assessed the immune response only through the spleen, excluding organs like the thymus, gut-associated lymphoid tissues, and bone marrow. While the spleen provides ample immune cells for evaluating innate and adaptive responses, a broader assessment would enhance understanding. Third, we did not confirm monosodium urate (MSU) crystal formation in HUA rats, although elevated UA and IL-6 levels, reduced T cells, serum IL-10, and renal changes [9] suggest their possible presence. MSU crystals predominantly activate innate immunity, while soluble UA has a more nuanced role [26]. Fourth, the presence of uricase in rats, absent in humans, limits their natural susceptibility to HUA, and pharmacological induction may not fully replicate human pathology. Differences in immune cell subsets and responses between rats and human also constrain direct extrapolation. Nonetheless, the rat model offers controlled conditions to explore systemic and organ-specific immune mechanisms relevant to human pathology. Finally, given the strong link between gut dysbiosis and HUA, future studies should investigate how dietary interventions influence immune function and microbiota composition to guide clinical translation [27].

## 4. Materials and Methods

### 4.1. Animals and Study Design

Male Wistar rats (BioLASCO Taiwan Co., Taipei, Taiwan), initially weighing approximately 200 g (6 weeks old), were acclimated to the animal facility for one week. The facility was maintained at 22 °C with a 12:12 h light–dark cycle, and the rats had free access to water and a chow diet (5001 Laboratory Rodent diet, Labdiet^®^, Richmond, IN, USA).

After an overnight fast, the rats were randomly divided into six groups (*n* = 8 per group), consisting of a normal control group (CON) and five hyperuricemic groups (HUA, UCP, UCS, USP, and USS). The CON group was fed with a high-fat (30% of total calories from fat), semi-purified powder diet (AIN-93M), which comprised 46.6 g maize starch, 15.5 g sucrose, 14 g casein, 14 g maize oil, 5 g cellulose, 3.5 g AIN-93M mineral mix, 1 g AIN-93 vitamin mix, 0.25 g choline bitartrate, 0.18 g cysteine, and 0.0008 g t-butylhydroquinone per 100 g of diet. The five hyperuricemic groups were fed modified diets (Table 4) containing either casein or soy protein isolate (MP Biomedicals, Solon, OHA) as protein sources, and maize oil, safflower-seed oil, or palm oil (Sigma-Aldrich Co., St Louis, MO, USA) as fat sources, along with 15 g of sucrose or 10 g of sucrose plus 2 g of OA and 3 g of UA. All diets were nutritionally balanced, providing equivalent amounts (*w*/*w*) of protein (14%), fat (14%), maize starch (46.6%), fiber (5%), minerals, and vitamins.

The five hyperuricemic groups were fed diets containing 2% OA (*w*/*w*) and 3% UA (*w*/*w*). Rats in the HUA, UCP, and UCS groups received casein as the protein source, with maize oil, palm oil, and safflower-seed oil as the fat sources, respectively. In contrast, rats in the USP and USS groups were fed diets with soy protein isolate as the protein source, and palm oil and safflower-seed oil as the fat sources. Rats fed OA and UA developed significant hyperuricemia, renal injury, and intratubular crystal deposition, as described in the studies by Mazzali et al. and Lo et al. [9,28].

All rats were pair-fed with the HUA group from week 1 to week 8, with body weight recorded biweekly. At the end of the 8th week, the rats were anesthetized with ketamine (150 mg/kg) and xylazine (15 mg/kg) and subsequently euthanized via cardiac puncture under anesthesia. The order of euthanasia was randomized among the groups. Blood samples were collected and separated into whole blood, serum, and plasma for subsequent analysis. Additionally, the spleens were dissected, weighed, and processed for further analyses.

The animal use protocol was reviewed and approved by the Institutional Animal Care and Use Committee (IACUC) of Changhua Christian Hospital, Changhua, Taiwan (approval #CCH-95005).

### 4.2. Blood Leukocyte Counts, Serum Biochemistry Parameters, and Plasma Cytokines

White blood cell (leukocyte) counts in the whole blood were determined using a hematology analyzer (GEN-S System 2, Beckman Coulter Inc., Miami, FL, USA). Serum biochemical parameters, including glucose, albumin, triglycerides, and UA, were measured using an automatic analyzer (Hitachi 7150, Hitachi, Ltd., Tokyo, Japan).

Plasma concentrations of TNF-α, IFN-γ, IL-6, IL-4, and IL-10 were quantified using commercially available enzyme-linked immunosorbent assays (ELISA; R&D Systems Inc., Minneapolis, MN, USA). All samples were analyzed in duplicate.

### 4.3. Immunocyte Subpopulations in the Peripheral Blood Leukocytes (PBLs) and Splenocytes

Fifty microliters of whole blood were incubated with specific antibodies targeting cell-surface antigens for 30 min at room temperature in the dark. The cell-surface markers included CD3-fluorescein isothiocyanate (FITC, clone G4.18), CD3-phycoerythrin (PE, clone G4.18), CD4-PE (clone OX-35), and CD8b-FITC (clone 341) for T cells; CD45RA-PE (clone OX-33) and IgM-FITC (clone G53-238) for B cells; CD11b/c-PE (clone OX-42) for monocyte/macrophage; HIS48 for granulocytes-FITC (clone HIS48); and NKR-P1A-PE (clone 10/78) for natural-killer cells. FITC-conjugated mouse IgG1 (clone A112-2) and PE-conjugated mouse IgG3 (clone A112-3) were used as nonspecific isotype-control antibodies. Following incubation, red blood cells were lysed using NH4Cl lysis buffer, and the peripheral blood leukocytes (PBLs) were washed twice in phosphate-buffered saline (PBS) containing 2% fetal bovine serum (FBS), and then resuspended in 500 μL PBS with 1% paraformaldehyde and 0.1% NaN3 for flow cytometry analysis.

Single-cell suspensions of splenocytes were prepared by mechanically dissociating the spleen tissue and passing the cells through a 100-mesh screen to remove capsular material and cellular debris. Red blood cells were lysed from the splenocytes using lysis buffer. The single-cell suspensions were washed twice in PBS with 2% FBS, and splenocytes (1 × 10^6^ cells) were labeled with specific antibodies.

**Figure 6:**
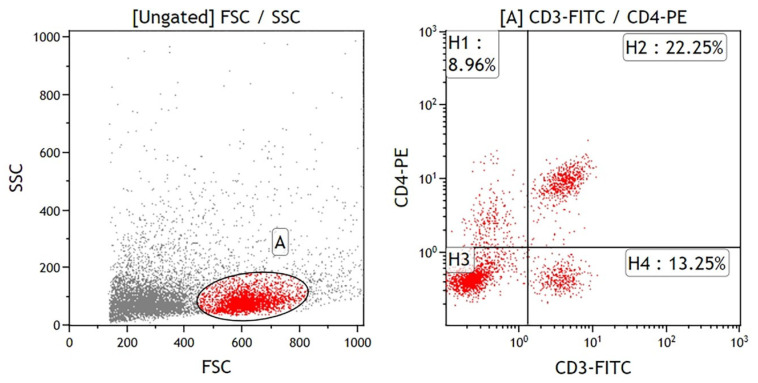
Flow cytometry analysis of lymphocyte populations. (**Left** panel) Forward scatter (FSC) versus side scatter (SSC) dot plot showing ungated cell populations. The gated population (region A, highlighted in red) represents lymphocyte-like cells based on size and granularity characteristics. (**Right** panel) Dot plot of CD3-FITC versus CD4-PE fluorescence intensities, gated on the population from region A. Quadrants H1, H2, H3, and H4 indicate subsets of cells expressing specific markers: H1 (CD3−CD4+), H2 (CD3+CD4+), H3 (CD3−CD4−), and H4 (CD3+CD4−). Percentages reflect the proportion of events within each quadrant relative to the gated population.

Immunofluorescence detection of PBLs and splenocyte subpopulations was performed using a Becton-Dickinson FASCScan flow cytometer with excitation capabilities at 488 nm, measuring FITC- and PE-conjugated mouse anti-rat monoclonal antibodies (BD Biosciences, San Jose, CA, USA). An example of the CD3 and CD4 splenocytes gating strategy is shown in Figure 6. The lymphocyte population was first gated based on forward scatter (FSC) and side scatter (SSC) characteristics (Figure 6, left panel). This initial gating (region A) was designed to exclude debris and non-lymphocyte populations by selecting cells with size and granularity typical of lymphocytes. Subsequently, the gated lymphocyte population was analyzed for CD3 and CD4 expression to identify T cell subsets (Figure 6, right panel). Quadrants were defined to differentiate CD3+CD4+ T cells (quadrant H2, 22.25%), CD3+CD4− T cells (quadrant H4, 13.25%), CD3−CD4+ cells (quadrant H1, 8.96%), and double-negative cells (quadrant H3). To ensure gating accuracy, FITC-conjugated mouse IgG1 and PE-conjugated mouse IgG3 were used as nonspecific isotype-control antibodies to set fluorescence thresholds and confirm specific staining of CD3 and CD4 markers.

### 4.4. Immunocyte Proliferation of Splenocytes

The in vitro cell proliferation of splenocytes in response to Con A, a T-cell mitogen, and LPS, a B-cell and monocyte/macrophage mitogen, was assessed. Splenocytes (2.5 × 10^5^ cells) were cultured in triplicate in RPMI 1640 medium, with or without the addition of Con A (5 μg/mL) and LPS (10 μg/mL), and incubated at 37 °C in a 5% CO_2_ atmosphere for 36 h. Cell proliferation was quantified using the colorimetric MTS assay (CellTiter 96^®^ AQueous one solution, Promega, Madison, WI, USA). The stimulation index of cell proliferation was calculated by dividing the absorbance of 490 nm of splenocytes cultured with RPMI-1640 medium and mitogen by the absorbance of those cultured with RPMI-1640 medium alone.

### 4.5. Cytokine Production of PBLs and Splenocytes

Four microliters of whole blood, collected in EDTA tubes, were treated with 36 mL of lysis buffer for 3 min at room temperature in the dark. The samples were then washed twice with PBS containing 2% FBS and resuspended in 4 mL of RPMI 1640 medium to isolate PBLs. PBLs (1 mL) and splenocytes (5 × 10^6^ cells/mL) were cultured with medium (spontaneous production), Con A (5 μg/mL, a mitogen for T cells), or LPS (10 μg/mL, a mitogen for monocytes/macrophages and B cells) at 37 °C in a 5% CO_2_ atmosphere for 18 h. Following incubation, supernatants were collected by centrifugation and stored at −80 °C. Cytokine concentrations in the supernatants were quantified using the ELISA method (DuoSet, R&D System, Minneapolis, MN, USA). All samples were analyzed in duplicate within a single assay.

### 4.6. Statistical Analysis

Data are presented as mean ± SEM. Group differences were evaluated using a one-way ANOVA with the SAS general linear model program (SAS Version 9.4, SAS Institute Inc., Cary, NC, USA). When ANOVA indicated a significant overall effect (*p* < 0.05), the protective least significant difference (LSD) test was used for post hoc comparisons between groups. Additionally, a two-way ANOVA was conducted to assess the main effects of protein and oil, as well as their interaction, on each parameter among the UCP, UCS, USP, and USS groups. A post hoc power analysis was performed using G*Power 3.1 to ensure this study was adequately powered [29]. For the five hyperuricemic groups, a sample size of seven to eight rats per group was required to achieve ≥80% power at a 5% significance level.

## 5. Conclusions

**Figure 7:**
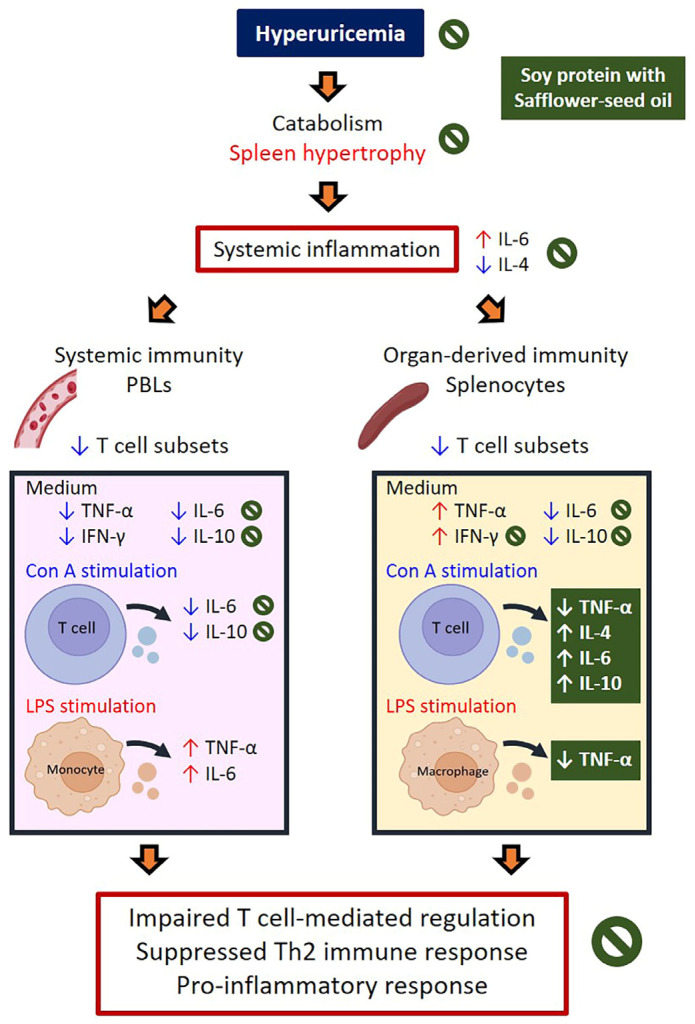
Schematic summarizing the effects of plant-based diet incorporating soy protein and safflower-seed oil on immune responses in HUA rats.

This study demonstrates that HUA, induced by OA and UA, leads to systemic inflammation and immune dysfunction, characterized by reduced T cell populations in PBLs and splenocytes, elevated leukocyte counts, and altered cytokine profiles under mitogen stimulation. Dietary interventions showed distinct effects on immune regulation: soy protein improved TNF-α and IL-10 production in PBLs and splenocytes, mitigating HUA-associated immune disruptions, while safflower-seed oil exhibited anti-inflammatory properties by reducing leukocyte counts, lowering plasma IL-6, increasing plasma IL-4 and T-suppressor splenocytes, and modulating cytokine production (TNF-α, IL-6, and IL-10). In contrast, casein and palm oil were linked to pro-inflammatory effects, with elevated plasma IL-6 and decreased IFN-γ. These findings suggest that a plant-based diet incorporating soy protein and safflower-seed oil may effectively manage HUA by reducing inflammation and enhancing Th2-mediated immune responses (Figure 7), underscoring the therapeutic potential of dietary strategies for immune dysfunction in HUA.

## Figures and Tables

**Table 1:** Serum biochemical values, leukocyte and splenocyte numbers, and spleen weights ^1^.

Group	Albumin	Glucose	Triglyceride	Uric Acid	Leukocytes	Splenocytes	Spleen Weight
	g/100 mL	mmol/L	mg/100 mL	mg/100 mL	10^3^/μL	×10^7^ Cells	g/kg
CON	4.05 ± 0.09	7.64 ± 0.26	121.1 ± 5.1	2.83 ± 0.08	3.84 ± 0.28	4.32 ± 0.57	1.88 ± 0.07
HUA	3.72 ± 0.08 *	7.94 ± 0.38	118.1 ± 5.9	4.29 ± 0.56 *	5.88 ± 0.63 *	3.58 ± 0.49	2.54 ± 0.11 *
UCP	3.55 ± 0.07	8.40 ± 0.23	150.3 ± 7.8 †	5.01 ± 0.42	6.29 ± 0.20	3.16 ± 0.31	2.62 ± 0.15
UCS	3.83 ± 0.09	7.68 ± 0.14	92.6 ± 2.2 †	3.18 ± 0.24 †	5.01 ± 0.40	3.60 ± 0.42	2.27 ± 0.18
USP	3.96 ± 0.11	8.44 ± 0.28	138.1 ± 8.7 †	4.30 ± 0.43	5.50 ± 0.41	3.37 ± 0.35	2.66 ± 0.15
USS	4.19 ± 0.06 †	7.82 ± 0.17	86.3 ± 2.6 †	2.98 ± 0.24 †	5.18 ± 0.42	3.72 ± 0.54	2.40 ± 0.08
Main effects for four groups (two-way ANOVA) ^2^							
Protein	<0.001	NS	NS	NS	NS	NS	NS
Oil	0.012	0.004	<0.001	<0.001	0.045	0.049	0.044
Interaction	NS	NS	NS	NS	NS	NS	NS

^1^ Values are means ± SEM, *n* = 8 per group. *, HUA vs. CON; †, UCP, UCS, USP, and USS vs. HUA (one-way ANOVA with least significant difference, *p* < 0.05). ^2^ Values of a two-way ANOVA are *p*-values for main effects and interactions of protein (casein or soybean protein) and oil (palm or safflower-seed oil) in the UCP, UCS, USP, and USS groups. NS, not significant.

**Table 2:** Percentages of lymphocytes, monocytes, granulocytes, and natural killer cells in peripheral blood leucocytes ^1^.

Group	Total CD3+	CD3+CD4+	CD3+CD8b+	CD45RA+IgM+	CD11b/c+GN−	CD11b/c+GN+	NKR+CD3−
	%	%	%	%	%	%	%
CON	62.9 ± 2.0	45.9 ± 1.8	15.0 ± 1.1	11.5 ± 0.9	6.53 ± 0.93	78.9 ± 4.2	5.48 ± 0.50
HUA	51.2 ± 2.0 *	38.2 ± 2.0 *	11.9 ± 0.6 *	18.0 ± 1.6 *	6.40 ± 0.67	62.2 ± 7.2	3.95 ± 0.42
UCP	57.0 ± 1.8	41.4 ± 1.3	10.5 ± 1.0	16.8 ± 2.4	6.60 ± 0.87	69.5 ± 8.6	3.69 ± 0.47
UCS	57.2 ± 1.8	41.3 ± 2.0	12.0 ± 1.2	10.6 ± 1.6 †	8.76 ± 0.76 †	52.4 ± 8.5	4.38 ± 0.57
USP	51.6 ± 2.0	36.9 ± 1.3	12.2 ± 0.9	13.0 ± 1.3 †	7.04 ± 0.67	36.6 ± 5.8 †	4.27 ± 0.38
USS	56.5 ± 2.6	41.3 ± 2.1	13.6 ± 0.7	10.5 ± 1.1 †	6.89 ± 0.73	31.6 ± 7.3 †	4.80 ± 0.47
Main effects for 4 groups (two-way ANOVA) ^2^							
Protein	NS	NS	NS	NS	NS	<0.001	NS
Oil	NS	NS	NS	0.011	0.029	NS	NS
Interaction	NS	NS	NS	NS	0.013	NS	NS

^1^ Values are means ± SEM, *n* = 8 per group. *, HUA vs. CON; †, UCP, UCS, USP, and USS vs. HUA (one-way ANOVA with least significant difference, *p* < 0.05). ^2^ Values of a two-way ANOVA are *p*-values for main effects and interactions of protein (casein or soybean protein) and oil (palm or safflower-seed oil) in the UCP, UCS, USP, and USS groups. CD3+, total T cells; CD3+CD4+, helper T cells; CD3+CD8b+, cytotoxic T cells; CD45RA+IgM+, mature B cells; CD11b/c+GN−, monocytes; CD11b/c+GN+, granulocytes; NKR+CD3−, natural killer cells; NS, not significant.

**Table 3:** Percentages of lymphocytes, macrophages, granulocytes, and natural killer cells in splenocytes ^1^.

Group	Total CD3+	CD3+CD4+	CD3+CD8b+	CD45RA+IgM+	CD11b/c+GN−	CD11b/c+GN+	NKR+CD3−
	%	%	%	%	%	%	%
CON	40.0 ± 2.5	20.1 ± 1.5	13.8 ± 1.4	23.7 ± 3.0	8.99 ± 0.80	8.84 ± 0.30	3.17 ± 0.27
HUA	32.6 ± 2.9 *	20.9 ± 2.6	8.5 ± 0.8 *	32.5 ± 3.5	8.49 ± 1.01	9.61 ± 0.47	2.94 ± 0.48
UCP	35.0 ± 2.7	23.5 ± 1.5	12.3 ± 0.6	27.4 ± 1.9	6.77 ± 0.57	9.91 ± 1.00	1.71 ± 0.23 †
UCS	33.2 ± 2.6	28.2 ± 3.2	14.3 ± 1.1	28.0 ± 3.1	9.63 ± 0.78	11.10 ± 1.22	3.31 ± 0.58
USP	28.2 ± 2.0	17.0 ± 1.4	9.8 ± 0.9	32.7 ± 2.1	9.12 ± 0.89	10.75 ± 0.25	1.86 ± 0.22 †
USS	41.0 ± 3.2 †	22.0 ± 1.4	15.7 ± 2.0 †	32.4 ± 2.2	4.13 ± 1.04 †	12.31 ± 1.25 †	2.11 ± 0.31
Main effects for 4 groups (two-way ANOVA) ^2^							
Protein	NS	NS	NS	0.047	NS	NS	NS
Oil	NS	NS	0.005	NS	NS	NS	0.012
Interaction	0.013	NS	NS	NS	<0.001	NS	NS

^1^ Values are means ± SEM, *n* = 8 per group. *, HUA vs. CON; †, UCP, UCS, USP, and USS vs. HUA (one-way ANOVA with least significant difference, *p* < 0.05). ^2^ Values of a two-way ANOVA are *p*-values for main effects and interactions of protein (casein or soybean protein) and oil (palm or safflower-seed oil) in the UCP, UCS, USP, and USS groups. CD3+, total T cells; CD3+CD4+, helper T cells; CD3+CD8b+, cytotoxic T cells; CD45RA+IgM+, mature B cells; CD11b/c+GN−, macrophage; CD11b/c+GN+, granulocytes; NKR+CD3−, natural killer cells; NS, not significant.

**Table 4:** Nutrition composition of experimental diets ^1^.

Ingredient/Group	CON	HUA	UCP	UCS	USP	USS
Protein type	casein	casein	casein	casein	soy protein	soy protein
Protein amount	14.0	14.0	14.0	14.0	14.0	14.0
Oil type	corn oil	corn oil	safflower-seed	palm oil	safflower-seed	palm oil
Oil amount	14.0	14.0	14.0	14.0	14.0	14.0
Corn starch	46.5692	46.5692	46.5692	46.5692	46.5692	46.5692
Sucrose	15.5	10.5	10.5	10.5	10.5	10.5
Cellulose	5.0	5.0	5.0	5.0	5.0	5.0
AIN 93M Mineral Mix	3.5	3.5	3.5	3.5	3.5	3.5
AIN 93 Vitamin Mix	1.0	1.0	1.0	1.0	1.0	1.0
Choline bitartrate	0.25	0.25	0.25	0.25	0.25	0.25
L-cystine	0.18	0.18	0.18	0.18	0.18	0.18
TBHQ	0.0008	0.0008	0.0008	0.0008	0.0008	0.0008
Oxonic acid	0	2.0	2.0	2.0	2.0	2.0
Uric acid	0	3.0	3.0	3.0	3.0	3.0

^1^ Modified-purified AIN-93M diet provides per gram: 4.11 kcal, 3.54 nonprotein kcal, 0.142 g protein, and 0.14 g fat. The diet provides 13.8% calories as protein, 55.5% calories as CHO, and 30.7% calories as fat. AIN, American Institute of Nutrition; TBHQ, tert-butylhydroquinone.

## Data Availability

All the data generated or analyzed during this study are included in this published article. The raw data are available from the corresponding author upon reasonable request.
